# Supplementary material for: Exploring Dry-Film FTIR Spectroscopy to Characterize Milk Composition and Subclinical Ketosis throughout a Cow’s Lactation
Source: Foods. 2021 Aug 29;10(9):2033. doi: 10.3390/foods10092033 (PMC8472635; doi:10.3390/foods10092033)
Supplement: Supplementary file 1 [file foods-10-02033-s001.zip › foods-1286374-supplementary.pdf]

foods

MDPI

Article

Exploring dry-film FTIR spectroscopy to characterize milk composition and subclinical ketosis Throughout a cow's lactation

Amira Rachah, Olav Reksen, Valeria Tafintseva, Felicia Judith Marie Stehr, Elling-Olav Rukke, Adam Martin, Achim Kohler and Nils Kristian Afseth

SUPPLEMENTARY FILE

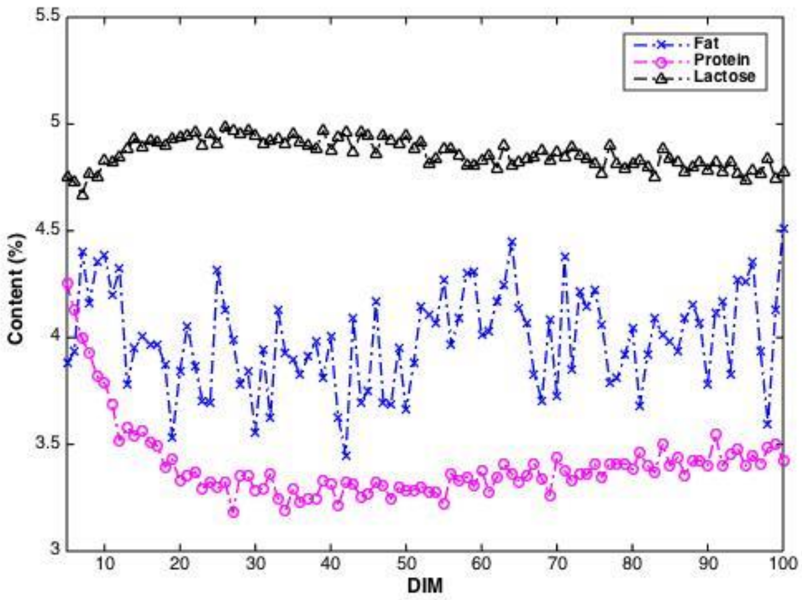

Figure S1. Corresponding plots of average chemical composition between DIM 5 and DIM 100

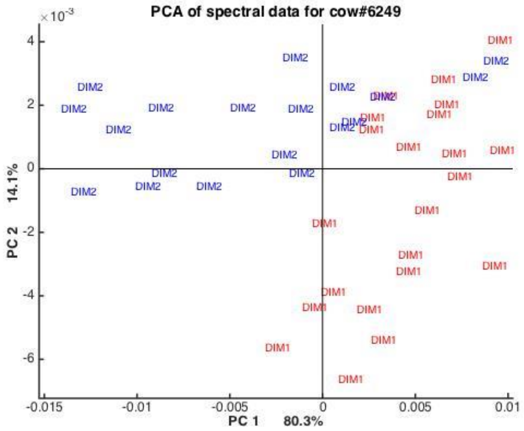

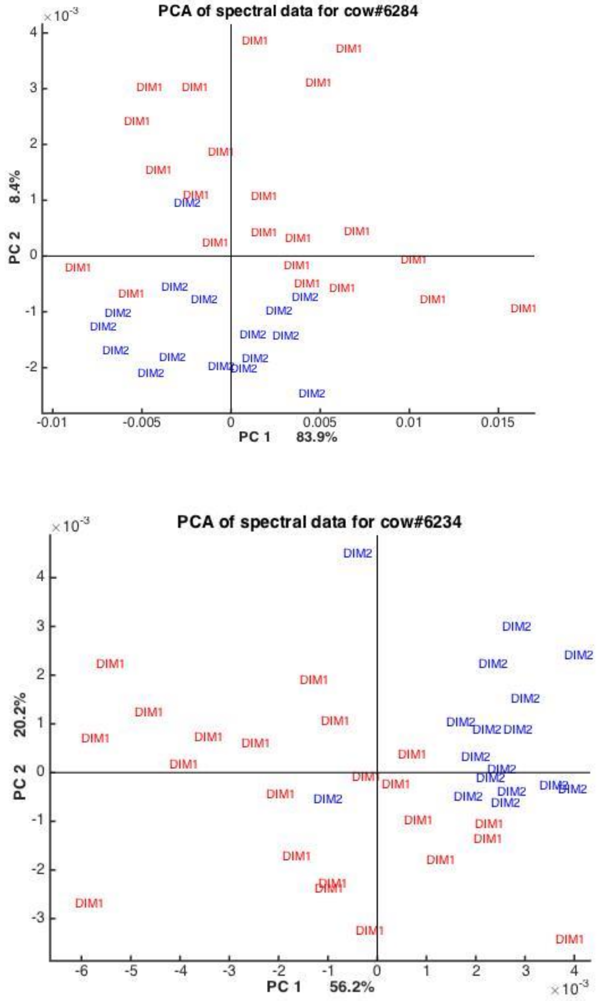

Figure S2. Score plots from PCA of FTIR spectra of milk from 3 randomly selected cows: DIM 1 (red color): 5 ≤ DIM ≤ 50 ; DIM 2 (blue color): 50 < DIM ≤ 100.

Table S1. Calibration and prediction results for fatty acid composition in milk samples. Data set A: 219 FTIR spectra from a previously published study were FTIR were used to calibrate for fatty acid contents in milk; and data set B: 25 milk samples randomly selected from the current study.

Fatty acid features	Calibration results (data set A+B; n=244; segmented CV)			Prediction results (data set B; test set size: 25 samples)		
	RMSECV	R^2	PLS factors	RMSEP	R^2	PLS factors
C10:0	0.14	0.94	11	0.16	0.91	16
C14:0	0.43	0.85	9	0.45	0.72	6
C16:0	0.80	0.92	11	1.20	0.74	17
C18:0	0.52	0.87	14	0.70	0.63	14
C18:1cis-9	0.55	0.96	10	0.64	0.91	10
CLA	0.08	0.83	16	0.10	0.32	1
SFA	0.75	0.96	10	0.96	0.88	14
MUFA	0.55	0.97	10	0.88	0.85	10
PUFA	0.15	0.77	18	0.18	0.32	5

Table S2. Classification results: all 2329 samples classified by SPLSDA model as DIM1 vs DIM2. Number of spectra as well as percent of samples classified are provided. Overall mode accuracy = 78.0%.

Predicted class / True class	DIM1		DIM2
DIM1 (Total=1289)	1000, 78%		289, 22%
DIM2 (Total=1040)	223, 21%		817, 79%

Table S3. Classification results: 895 samples of PAR1 group classified by SPLSDA model as DIM1 vs DIM2. Number of spectra as well as percent of samples classified are provided. Overall mode accuracy = 79.3%.

Predicted class / True class	DIM1	DIM2
DIM1 (Total=486)	375, 77%	111, 23%
DIM2 (Total=409)	74, 18%	335, 82%

Table S4. Classification results: 608 samples of PAR2 group classified by SPLSDA model as DIM1 vs DIM2. Number of spectra as well as percent of samples classified are provided. Overall mode accuracy = 74.3%.

Predicted class / True class	DIM1	DIM2
DIM1 (Total=334)	239, 72%	95, 28%
DIM2 (Total=274)	61, 22%	213, 78%

Table S5. Classification results: 826 samples of PAR>2 group classified by SPLSDA model as DIM1 vs DIM2. Number of spectra as well as percent of samples classified are provided. Overall mode accuracy = 81.0%.

Predicted class / True class	DIM1	DIM2
DIM1 (Total=469)	388, 83%	81, 17%
DIM2 (Total=357)	76, 21%	281, 79%
